# Current Perspectives on Immunotherapy in the Peri-Operative Setting of Muscle-Infiltrating Bladder Cancer

**DOI:** 10.3389/fonc.2020.568279

**Published:** 2020-10-21

**Authors:** Paolo Andrea Zucali, Nadia Cordua, Federica D'Antonio, Federica Borea, Matteo Perrino, Fabio De Vincenzo, Armando Santoro

**Affiliations:** ^1^Department of Medical Oncology and Hematology, Humanitas Clinical and Research Center—Istituto di Ricovero e Cura a Carattere Scientifico (IRCCS), Rozzano, Italy; ^2^Department of Biomedical Sciences, Humanitas University, Milan, Italy

**Keywords:** muscle-infiltrating bladder cancer (MIBC), immunotherapy, neoadjuvant, adjuvant, predictive biomarkers

## Abstract

Patients with muscle-infiltrating bladder cancer (MIBC) present a high risk of postoperative recurrence and death from metastatic urothelial cancer despite surgical resection. Before the use of peri-operative chemotherapy, about half (52%) of patients undergoing radical cystectomy had had a relapse of tumor disease within 5 years of surgery. However, when peri-operative cisplatin-based chemotherapy is added to radical cystectomy for patients with MIBC it provides limited benefit in terms of survival, disease recurrence and development of metastases, at the expense of toxic effects. In fact, a significant proportion of patients still recurs and die to metastatic disease. Given the success of immune-oncological drugs in metastatic urothelial cancer, several trials started to test them in patients with non-metastatic MIBC either in neo-adjuvant and adjuvant setting. The preliminary results of these studies in neo-adjuvant setting are showing great promise, confirming the potential benefits of immunotherapy also in patients with non-metastatic MIBC. The aim of this review is to present an overview of developments happening on the introduction of immunotherapy in peri-operative setting in non-metastatic urothelial cancer. Moreover, an analysis of the critical issues regarding how best customize the delivery of immunotherapy to optimize efficacy and minimize the adverse effects, with particular focus on potential prognostic and predictive molecular biomarkers, is done.

## Introduction

The urothelial carcinoma of the urinary tract is one of the most prevalent cancer in the world and the urinary bladder is the most frequent pathologic site of occurrence (1).

Approximately 30% of patients with bladder cancer have a MIBC, namely pT2 or more according to TNM staging (2). Current guidelines strongly recommend a combined approach of radical cystectomy (RC) and peri-operative cisplatin-based chemotherapy, especially in the neoadjuvant setting, for patients with MIBC (3, 4, 4–8). The survival benefit of neoadjuvant chemotherapy (NAC) was mainly correlated with the pathologic complete response (pCR), that means to be cancer free (pT0N0M0) at the time of RC. The prognostic value of pCR is meaningful because several studies have correlated the degree of pathologic response with survival after RC and the achievement of pCR indicates the best chance of long-term survival. In a meta-analysis of 13 trials (886 patients analyzed after NAC and RC), the pathological complete response (pCR) rate was 28.6% and patients who achieved pCR (pT0N0M0 stage) after NAC had a better OS (relative risk for OS was 0.45, *p* < 0.00001) and relapse free survival (RFS) (relative risk for RFS was 0.19, *p* < 0.00001) (9). Unfortunately, neoadjuvant cisplatin-based chemotherapy is still underused. In fact, only 20% of eligible patients are treated with NAC. Approximately 50% of patients were ineligible to receive cisplatin for pre-existing comorbidity (such as kidney failure with a creatinine clearance less than 60 mL/min; hearing loss of grade 2 or more; impaired performance status; neuropathy of grade 2 or higher; cardiac dysfunction) while the remaining 30% refused the treatment (10). These cisplatin-unfit patients could be treated with carboplatin and gemcitabine, but there are no literature data to support this strategy.

It has been shown that urothelial carcinoma is immune responsive. Several literature data demonstrated how the Bacillus Calmette-Guerin (BCG) instillations impact on decreasing the risk of tumor recurrence and progression (11). Actually, the BCG instillations are standard of care for non-muscle invasive high-risk tumors after transurethral resection of the bladder (TURB) (12). Moreover, bladder tumor carries a high mutational load, which leads to the development of a high number of neoantigens, appearing as a good candidate for immunotherapy with checkpoint inhibitors (CPIs) (13).

In the last years, CPIs have been approved in the second-line setting for patients with metastatic bladder tumor who progressed during or after cisplatin-based chemotherapy (14–18). Moreover, atezolizumab and pembrolizumab have been already approved in first-line setting in patients ineligible for cisplatin and with positive tumor PD-L1 expression (19).

Currently, several trials on the efficacy and safety of CPIs for MIBC are in progress in peri-operative setting. The aim of this review is to present an overview of developments happening on the introduction of immunotherapy in peri-operative setting in non-metastatic urothelial cancer. Moreover, an analysis of the critical issues regarding how best customize the delivery of immunotherapy to optimize efficacy and minimize the adverse effects, with particular focus on potential prognostic and predictive molecular biomarkers, is done.

## Neadjuvant Immunotherapy

The success of CPIs in terms of response in the advanced/metastatic state of bladder cancer has supported ongoing clinical trials in the adjuvant and neoadjuvant settings in patients with non-metastatic MIBC. These clinical trials are testing CPIs as monotherapy or in combination with other class of CPIs or with chemotherapy or with target therapy. Tables 1–**4** summarize the most important neoadjuvant trials in MIBC.

**Table 1:** Overview of selected neoadjuvant trials in MIBC testing mono-immunotherapy.

**Single-agent therapy**					
**Drugs**	**Number of patients**	**Study design**	**Primary endpoint**	**Status**	**Trial ID**
Avelumab	10	Phase II	Change in T-cell subpopulations	Not yet recruiting	NCT03498196 (BL-AIR)
		Open Label			
		Single arm			
Atezolizumab	96	Phase II	pCR rate	Active, not recruiting	NCT02662309 (ABACUS)
		Open Label			
		Single arm			
Atezolizumab	20	Phase II	pCR rate	Recruiting	NCT03577132
		Open Label			
		Single arm			
Atezolizumab	42	Phase II	pCR rate	Active, not recruiting	NCT02451423
		Open Label			
Pembrolizumab	114	Phase II	pCR rate	Has results	NCT02736266 (PURE-01)
		Open Label			
Pembrolizumab	40	Phase II	pCR rate	Recruiting	NCT03212651 (PANDORE)
		Open Label			
		Single arm			

*ID, identification number; pCR, pathologic complete response*.

### Mono-Immunotherapy

Several clinical studies testing mono-immunotherapy are in progress. The PURE-01 trial (neoadjuvant pembrolizumab for MIBC) and the ABACUS trial (pre-operative atezolizumab in MIBC) are the first published studies reporting a complete response rate of 42 and 29%, respectively (20, 21).

The PURE-01 trial, a phase 2 single-arm study, enrolled 50 patients with MIBC to receive three cycles of pembrolizumab 200 mg every 3 weeks before RC (20). The inclusion criteria were urothelial histology, clinical stage cT3bN0 or lesser (evaluation with CT, MRI, or PET/CT), residual disease after TURBT and good general conditions (ECOG PS 0–1). Primary endpoint was pCR rate (pT0) at the time of surgery. Among the 50 patients enrolled, 27 (54%) had cT3 tumor, two (4%) cT2-3N1 tumor, and 21 (42%) cT2 tumor. Forty-six patients (92%) were eligible to receive cisplatin chemotherapy. All treated patients underwent RC. In all population, the pCR rate was 42% (21 out of 50 patients). Six patients had residual pTa, pTis, or pT1 stage tumor. Therefore, 27 patients (54%) resulted down staged to non-muscle invasive tumor after immunotherapy. Considering only the 35 patients with a PD-L1 combined positive score ≥10%, the pCR rate was 54%. Moreover, a significant non-linear association between tumor mutation burden (TMB) and pCR (pT0) was observed (cut off: 15 mutations/Mb) and the expression of several genes before the administration of pembrolizumab was significantly different between pT0 and non-pT0 cohorts. Finally, the analysis of post-therapy lesions compared with baseline lesions showed an overall increase in immune-gene expression, a lower TMB (5.7 mutations/Mb post-therapy vs. 11.0 mutations/Mb at baseline), and an increased PD-L1 combined positive score (although not significant: *p* = 0.1402). The most frequent adverse event (AE) was thyroid dysfunction in nine patients (18%) whereas only three patients (6%) experienced grade 3 AE. Pembrolizumab discontinuation was done in only one patient. Clavien Dindo grade > II postsurgical complications occurred in 15 patients (30%) whereas delayed immune-related AEs including pyrexia in 3 patients (6%), pruritus in 3 patients (6%), and xerostomia in 2 patients (4%) occurred within 2 months post-operatively. This protocol was emended to allow the enrollment of patients with predominant variant histology (VH), usually chemo-resistant and excluded from clinical trials (22). Overall, 114 patients were enrolled and 34 of them (30%) presented with VH, including 19 (17%) with predominant VH. In all population, the pCR rate (pT0) was 35%, whereas excluding predominant VH patients was 41%. On the one hand this study showed that patients harboring squamous-cell carcinoma or lymphoepithelioma-like variant feature had a higher pCR rate compared with other predominant VH, but on the other hand it observed that PD-L1 combined positive score and TMB are the key response predictors irrespective of the histological subtypes.

The ABACUS trial, a phase 2 single-arm study, investigated two cycles of atezolizumab (1,200 mg every 3 weeks) before RC in 69 patients with MIBC (T2-4N0M0) ineligible to cisplatin or who refused chemotherapy (21). The primary endpoint of the study was the pCR rate (pT0) at the time of surgery. At baseline, 53 patients (77%) had cT2 tumor, 11 (16%) cT3 tumor, and 5 (7%) cT4 tumor. Sixty-two patients (90%) underwent RC. The pCR rate was 29% (pT0 23%, Tis 6%) in all population and 40% in patients with high PD-L1 expression. Seven patients had residual pT1 stage tumor and therefore 39% of patients resulted down staged to non-muscle invasive tumor after immunotherapy. Notably, an increase of PD-L1 positivity from 35 to 73% was detected with exposure to atezolizumab. Grade 3 and 4 adverse events occurred in eight patients (11%) whereas Clavien Dindo grade > II postsurgical complications occurred in 12 patients (17%).

The pCR rate achieved by mono-immunotherapy in PURE-01 and ABACUS trials looks non-inferior to NAC (38% with accelerated MVAC regimen; 28.6% in a meta-analysis of 13 trials). However, the absence of long-term follow-up to assess the time of relapse and the impact on OS must induce us to be very cautious.

Luckily, many phase II trials are still testing mono-immunotherapy in neo-adjuvant setting in MIBC to corroborate its results.

Table 1 summarizes the most important neoadjuvant trials in MIBC testing mono-immunotherapy.

### Immune Combination Therapy

Many phase I and phase II trials are testing the combination of CTLA-4 and PD-L1 inhibitors in the neoadjuvant setting with the intent on one hand to improve the activity of immunotherapy in terms of a higher rate of pCR and a reduced risk of recurrence and death, and on the other hand avoiding that the potentially greater toxicity of the combination delays or precludes surgery. In patients with metastatic or advance urothelial carcinoma enrolled in CHECKMATE-032 trial, the combination of nivolumab 1 mg/kg plus ipilimumab 3 mg/kg achieved a higher response rate compared to nivolumab alone (38.0 vs. 25.6%) but with a higher incidence of grade 3–4 treatment-related adverse events (39.1 vs. 26.9%).

Table 2 gives an overview of ongoing selected neoadjuvant trials testing immune combination therapy.

**Table 2:** Overview of selected neoadjuvant trials in MIBC testing immune combination therapy.

**Immune-combination therapy**					
**Drugs**	**Number of patients**	**Study design**	**Primary endpoint**	**Status**	**Trial ID**
Nivolumab + Ipilimumab	54	Phase Ib	Safety, pCR rate	Recruiting	NCT03387761 (NABUCCO)
		Open label			
Nivolumab +/– Ipilimumab	45	Phase II Open label	Number of RC within 60 days after neoadjuvant therapy	Recruiting	NCT03520491 (CA209-9DJ)
Durvalumab + Tremelimumab	54	Phase I Open label	Incidence of AEs determined by extreme toxicity	Active, not recruiting	NCT02812420
Durvalumab + Tremelimumab	6	Phase II Open label	ORR	Active, not recruiting	NCT03234153 (NITIMIB)
Durvalumab + Tremelimumab vs. GC or MVAC or PaGC	99	Phase II Open label	pCR rate	Recruiting	NCT03472274 (DUTRENEO)
Nivolumab +/– Urelumab	44	II Open label	Tumor infiltrating CD8+ T-cells density at RC	Recruiting	NCT02845323

*ID, identification number; pCR, pathologic complete response; RC, radical cystectomy; AEs, adverse events; ORR, overall response rate; GC, gemcitabine, cisplatin; MVAC, methotrexate, vinblastine, doxorubicin, and cisplatin; PaGC, paclitaxel, gemcitabine, cisplatin*.

The results of these trials are needed to confirm the efficacy of the combination of CPIs, to define the best combination, the management of immune-related AEs, the duration of treatment, which patients have more benefits, and evaluation of cost-effectiveness.

### Chemo-Immunotherapy Combinations

The combination of chemotherapy with immune CPIs in urothelial cancer has the aim to improve the efficacy of both treatment strategies with a synergistic effect and to enlarge the range of patients who benefit of pharmacological therapy (23). In fact, chemotherapy could modify the tumor microenvironment composition, on one hand increasing the tumor infiltration by lymphoid cells, myeloid cells, and CD8 T-cells but on the other hand decreasing the tumor infiltration by regulatory T-cells and myeloid-derived suppressive cells. Moreover, chemotherapy induces immunologic cell death that augments the tumor antigens presentation through Major histocompatibility complex I (MHC-I) (24). In non-small-cell lung cancer (NSCLC) patients, the combination of cisplatin-based chemotherapy and immune CPIs has become standard of care (25). Unfortunately, a single arm phase II trial testing a combination regimen of GC and ipilimumab in patients with urothelial cancer failed to achieve its primary endpoint of a one-year OS of >60% (26).

Currently, many studies have been exploring the effectiveness of the combination of chemotherapy with immune CPIs in neoadjuvant setting.

Among others, one is evaluating neoadjuvant Pembrolizumab in combination with chemotherapy. This (NCT02365766) is a two-part trial with a one-arm phase Ib portion followed by a two-arm phase II portion designed to enroll overall 83 patients with MIBC or urothelial cancer (T2-4aN0M0) who are candidates for neoadjuvant therapy. The cisplatin-eligible patients are treated with the combination GC + pembrolizumab whereas the cisplatin-ineligible patients are treated with gemcitabine + pembrolizumab. The study treatment is stratified into two cohorts based on cisplatin eligibility. The primary endpoint of the phase Ib portion is the definition of safety and tolerability of the combination. The primary endpoint of phase 2 portion is the rate of pathologic muscle invasive response. Preliminary data analysis for 40 evaluable patients were presented in ESMO 2018. No dose limiting toxicities were observed in 6 pts on phase 1b. Only one patient did not have RC due to AE. The pathologic non-muscle invasive rate was 60% (baseline stage was cT2 51%, cT3 44%, cT4a 5%) and did not correlate with baseline PD-L1 score. (27).

Table 3 summarizes the most important neoadjuvant trials in MIBC testing chemo-immunotherapy combinations.

**Table 3:** Overview of selected neoadjuvant trials in MIBC testing chemo-immuntherapy combinations.

**Chemo-immunotherapy combinations**					
**Drugs**	**Number of patients**	**Study design**	**Primary endpoint**	**Status**	**Trial ID**
Nivolumab + GC	43	Phase II Open Label Single arm	pCR rate	Has results	NCT03294304 (BLTASST-1)
Nivolumab + GC	76	Phase II Open Label Single arm	pCR rate 2 years MFS	Active, not recruiting	NCT03558087
Pembrolizumab + GC	39	Phase II Open Label Single arm	pD rate (< pT2)	Active, not recruiting	NCT02690558
Pembrolizumab + GC or G	83	Phase I/II	Safety pMI-RR	Recruiting	NCT02365766
Avelumab vs. Avelumab + MVAC vs. Avelumab + CG vs. Avelumab + PaG	166	II Open Label Single arm	pCR rate	Recruiting	NCT03674424 (AURA)
Durvalumab + MVAC vs. Durvalumab + Tremelimumab + MVAC	120	I/II non-comparative, open-label	pCR rate	Recruiting	NCT03549715 (NEMIO)

*ID, identification number; GC, gemcitabine, cisplatin; pCR, pathologic complete response; 2 yrs MFS: two years metastasis-free survival; pD, pathologic down-staging; G, gemcitabine; pMI-RR, pathologic muscle invasive response rate; MVAC, methotrexate, vinblastine, doxorubicin, and cisplatin; PaG, paclitaxel, gemcitabine*.

### Immunotherapy Combined With Novel Therapeutic Agents

The effects of immunotherapy may be enhanced by the combination with several novel agents with different mechanism of action. For example, tumorigenesis is a process that involves both angiogenesis and immunosuppression in the tumor microenvironment and targeting both pathways simultaneously may enhance the antitumor capabilities of the immune system (28). Also the gene-targeted therapies may accentuate the anti-tumor response of immunotherapy through upregulated immune-mediated killing and inhibition of tumor-mediated immunosuppression (29). Moreover, vaccines may also play a role in immunotherapeutic strategies for urothelial cancer. Though these new strategies are under evaluation in metastatic setting, some studies are ongoing also in the neoadjuvant setting.

The NEODURVARIB trial (NCT03534492) is a single-arm phase II study designed to assess the activity and safety of the combination of durvalumab and olaparib as neoadjuvant treatment in 29 patients with resectable MIBC (cT2-T4a). Olaparib is an inhibitor of the enzyme poly ADP ribose polymerase (PARPi). The PARPis usually amplify the DNA damage increasing the tumor mutational burden and consequently making tumors more immunogenic by increasing neoantigen production and upregulating the PD-L1 expression. Durvalumab 1,500 mg is delivered every 4 weeks for up to 2 months (2 cycles) and olaparib 150 mg twice daily is administered for up to 56 days (2 cycles of 28 days each cycle). The primary endpoint of this study is to test the impact of this combination in the molecular profile of resectable MIBC in terms of pCR. Preliminary results show a pCR of 50% with a good toxicity profile (8.3% of patients experienced G3–4 drug related AEs) (30).

The ABATE trial (NCT04289779) is an open-label phase II study testing the activity of cabozantinib combined with atezolizumab in 42 patients with MIBC who are ineligible for cisplatin-based therapy or decline cisplatin-based therapy. Cabozantinib plays important roles in tumor cell proliferation and tumor neovascularization targeting MET, vascular endothelial growth factor receptor (VEGFR), AXL, and RET. Moreover, cabozantinib promotes tumor-immune suppression targeting TYRO3, MER, and AXL. The dose of atezoluzumab is 1,200 mg IV every 3 weeks (Day 1) plus Cabozatinib 40 mg orally daily (Day 1 through 21). Prior to RC, patients receive three cycles of therapy.

In a phase 2 trial (NCT01353222), the therapeutic feasibility of DN24-02 in HER2+ urothelial carcinoma patients who are at high risk of relapse following RC was investigated. DN24-02 is an autologous immunotherapeutic vaccine that generates an immune response against HER2/neu. Compared to patients who received standard of care surveillance, patients receiving the three infusion cycles showed and increased HER2 antibody responses, serum cytokines (IL-2, IFN-gamma, and TNF-alpha), *in vitro* IL-2 and IFN-gamma accumulation, and antigen-specific T cell responses. In subgroup analysis, patients with low tumor burden showed more favorable hazard ratios for OS. However, in all population DN24-02 failed to increase OS or recurrence-free survival (31).

Table 4 summarizes the most important neoadjuvant trials in MIBC testing immunotherapy combined with novel therapeutic agents.

**Table 4:** Overview of selected neoadjuvant trials in MIBC testing immunotherapy combined with novel therapeutic agents.

**Novel agents-immunotherapy combinations**					
**Drugs**	**Number of patients**	**Study design**	**Primary endpoint**	**Status**	**Trial ID**
Durvalumab + Olaparib	29	Phase II Open Label Sigle arm	pCR rate	Completed	NCT03534492 (NEODURVARIB)
Atezolizumab + Cabozantinib	42	Phase II Open Label Sigle arm	pRR rate	Not yet recruiting	NCT04289779 (ABATE)
Pembrolizumab + Epacadostat	38	Phase II Open Label Sigle arm	pCR rate	Not yet recruiting	NCT03832673

*ID, identification number; GC, gemcitabine, cisplatin; pCR, pathologic complete response; pRR, pathologic response rate*.

### Adjuvant Immunotherapy

The aim of the adjuvant therapy is to reduce the risk of relapse and improve OS by eliminating potential residual cancer cells after surgery. However, the role of adjuvant chemotherapy in urothelial cancers is still debatable because the available data supporting its use in clinical practice are scarce and not convincing (32–34). A recent meta-analysis of four trials including patients with locally advanced MIBC showed a pooled hazard ratio for PFS and OS across the studies of 0.48 (*p* < 0.00001) and 0.63 (*p* = 0.0009), respectively. Therefore, the absolute increases in PFS and OS for locally advanced MIBC were 17 and 10%, respectively (6).

The worse oncological outcomes for patients with residual cancer disease after RC gives an opportunity to evaluate newer agents or other strategies such as adjuvant immunotherapy. The role of immune CPIs in adjuvant setting has yet to be defined in several solid tumors. First studies evaluating CPIs in the adjuvant setting were performed in melanoma patients (35–37). Ipilimumab was first approved as adjuvant therapy in 2015 by Food And Drug Administration (FDA) whereas pembrolizumab and nivolumab have been recently approved by the European Medicines Agency (EMA) and the FDA for patients with stage III melanoma. Several phase III trials are evaluating the role of immune CPIs in the adjuvant setting in urothelial cancers. In some of these adjuvant trials, lack of response to NAC is a criterion of eligibility. They are comparing single-agent CPI with placebo or observation.

The IMvigor010 trail (NCT02450331) is an open-label, randomized, multicenter phase III study to evaluate the efficacy and safety of adjuvant treatment with atezolizumab compared to observation in high-risk muscle-invasive urothelial carcinoma of the bladder or upper urinary tract. In particular, eligible participants are patients treated with previous NAC and with post-surgical tumor stage of ypT2-4a or ypN+ or participants who have not received prior NAC and with post-surgical tumor stage of pT3-4a or pN+. The trial is active without recruitment: 809 participants have been randomized 1:1 into the atezolizumab group (16 cycles, up to 1 year) or into the control group. The primary endpoint is DFS, as assessed by the investigator. The primary analysis of this trial was recently presented. In the atezolizumab and observational arms, respectively, 48% and 47% had NAC; 7 and 6% had urothelial carcinoma of the upper urinary tract as primary disease; 48% each had disease at the lymph nodes level. Unfortunately, this phase III did not meet its primary endpoint of DFS. In fact, the median DFS was 19.4 (15.9, 24.8) months for patients treated with adjuvant atezolizumab compared to 16.6 (11.2, 24.8) months for patients in the observation arm [HR 0.89 (0.74, 1.08); *p* = 0.2446]. Moreover, more treatment discontinuation due to AEs was seen compared to metastatic urothelial cancer (38).

It is still unknown if tumors retain their sensitivity to the immunotherapy and to what extend adjuvant is needed to maintain tumor-specific T cell response (adjuvant maintenance therapy). Several trials are investigating the role of immunotherapy administered consecutively either in neoadjuvant and adjuvant setting.

The CA017-078 study (NCT03661320) is a randomized phase III trial (three arms) designed to compare neoadjuvant standard of care chemotherapy (GC) alone (Arm A) vs. nivolumab/BMS-986205 + chemotherapy (GC) followed by nivolumab/placebo + BMS-986205/placebo after RC (Arm B) vs. nivolumab/BMS-986205 + chemotherapy (GC) followed by nivolumab/BMS-986205 after RC (Arm C) in patients with MIBC (T2–T4aN0M0). BMS-986205 is a potent, selective, and orally bioavailable indoleamine 2,3-dioxygenase 1 (IDO1) inhibitor, with a potential antineoplastic and immunomodulating activities. The IDO1 is overexpressed by several tumor cell types and plays a key role in immunosuppression. By targeting and inhibiting IDO1 and decreasing kynurenine, BMS-986205 is able to determine a reduction in tumor-associated regulatory T cells (Tregs) and to restore and promote the activation and proliferation of several immune cells, such as natural killer cells, dendritic cells, and T lymphocytes cells.

Table 5 summarizes the most important adjuvant trials in MIBC testing immunotherapy.

**Table 5:** Overview of adjuvant trials in MIBC testing immunotherapy.

**Drugs**	**Number of patients**	**Study Design**	**Primary endpoint**	**Status**	**Trial ID**
**ADJUVANT IMMUNOTHERAPY**					
Pembrolizumab	739	Phase III Open Label	OS, DFS	Recruiting	NCT03244384 (AMBASSADOR)
Atezolizumab	809	Phase III Open Label	DFS	Active, non recruiting	NCT02450331 (IMvigor010)
Nivolumab	700	Phase III Open Label	DFS	Active, non recruiting	NCT02632409 (Checkmate 274)
NA Durvalumab + GC and A Durvalumab vs. NA GC	1050	Phase III Open Label	pCR rate, EFS	Recruiting	NCT03732677 (NIAGARA)
NA Pembrolizumab + GC and A Pembrolizumab vs. NA GC	790	Phase III Double-blind	pCR rate, EFS	Recruiting	NCT03924856 (KEYNOTE-866)
**NEOADJUVANT AND ADJUVANT IMMUNOTHERAPY**					
NA Nivolumab/BMS-986205 + GC and A Nivolumab/BMS-986205 vs. NA Nivolumab/BMS-986205 + GC and A Nivolumab/placebo + A BMS-986205/placebo vs. NA GC	1,200	Phase III Partially blinded	pCR rate, EFS	Recruiting	NCT03661320

*ID, identification number; OS, Overall survival; DFS, Disease Free Survival; NA, Neoadjuvant; GC, gemcitabine, cisplatin; A, Adjuvant; pCR, pathologic complete response; EFS, Event-Free Survival*.

## Discussion

Despite the robust data achieved by immunotherapy with CPIs in metastatic disease and the promising results in the peri-operative setting, several challenges still need to be addressed to further establish immunotherapy as a new standard of care in the management of patients with not metastatic MIBC.

The first issue is if the role of the pathological response on survival outcomes achieved with immune CPI therapies is comparable with that achieved with NAC. In fact, as in breast and rectal cancer, the pathologic downstaging got by NAC in bladder cancer is associated with increased OS. In a meta-analysis including 13 trials for a total of 886 patients analyzed after NAC and RC, the pathological complete response rate was 28.6% and patients who achieved pathological complete response in the primary tumor and the lymph nodes presented a relative risk for recurrence-free survival and for OS of 0.19 (*p* < 0.00001) and 0.45 (*p* < 0.00001), respectively (7). In the PURE-01 trial, 42% of not selected patients were pT0 after neo-adjuvant pembrolizumab whereas in the ABACUS trial, 31% of not selected patients achieved pathological complete response after neo-adjuvant atezolizumab (20, 21). However, the hypothesis that pathological complete response to immune CPI may portend a survival benefit, as with chemotherapy, remains unproven and needs to be confirmed.

The second issue regards the radiological tumor assessment that still needs to be standardized for immune CPIs therapies in neoadjuvant setting. Tumor lesions respond to immune CPIs with different modalities compared to chemotherapy, raising doubts about the interpretation of changes in tumor burden. The specific mechanism of action of immune CPIs, with an immune and T-cell activation, may lead to unusual patterns of response, namely pseudo-progression and hyperprogression. In case of pseudo-progression, the tumor initially increases in size but bit by bit stabilizes or responds to ongoing immunotherapy. This phenomenon is a consequence of an infiltration of immune cells within the tumor leading to a temporary increase in volume. Retrospective analyses showed that prevalence of the pseudoprogression in urothelial carcinoma ranges from 1.5 to 17% (39). Hyperprogression was first observed during immunotherapy in patients with melanoma and it is described as a rapid anomalous disease progression with a prevalence of 6% in solid tumors or lymphomas (40). The mechanisms underlying hyperprogression are still undefined (41). The clinical impact of psudoprogression and hyperprogression in neoadjuvant setting is yet to be defined.

The third issue regards the importance to not lose the opportunity to undergo definitive RC, the only therapeutic act that has proven to have a healing role in MIBC, because of complications or rapid progression. In the ABACUS trial, 3% of patients could not undergo RC due to immune CPI-related adverse events whereas in the PURE-01 trial, one out of 114 patients (0.9%) did not receive RC due to progression of disease (21, 22). Moreover, the preliminary data analysis for 40 evaluable patients enrolled in a phase Ib-II trial (NCT02365766) and treated with NAC + pembrolizumab reported that one patient did not have RC due to AE (27). A better selection of patients could avoid this potential risk.

The fourth issue regards to establish the criteria for the identification of patients who really benefit from immunotherapy in neoadjuvant setting, as cisplatin-based chemotherapy remains the standard for cisplatin-eligible patients. In fact, not all patients benefit from immunotherapy considering that response rates of immunotherapy ranges from 13 to 31% in unselected patients with metastatic urothelial carcinoma (42). In peri-operative management, distinguishing responder patients from non-responder ones in advance might allow to personalize immunotherapy use, by administering it only to those patients who really benefit most and, on the other hand, by directing non-responders to chemotherapy or immediate surgery, giving them the best chance of therapeutic success and minimizing unnecessary side effects and costs. Several attempts have been done to identify reliable biomarkers to predict the response to immune CPIs and to guide treatment decisions. Unfortunately, no one is yet available for clinical practice. The most important and promising predictive biomarkers under evaluation are PD-L1 expression, tumor mutational burden (TMB), immune cell gene expression profiling, CD8+ cells and Granzyme B, molecular subtyping (Table 6).

**Table 6:** Potential predictive biomarkers for immunotherapy with immune CPIs.

	**Rationale**	**Open problems**
PD-L1	Hypothesis that high levels of PD-L1 in tumor and/or immunological cells in tumor microenvironment may predict clinical response to CPIs with good evidence of correlation in NSCLC, melanoma, renal cell carcinoma	• Discordant results across different trials • Different IHC platforms, detection antibodies, cell types evaluated, and scores for defining positivity • Dynamic marker (variable over time and space) • No evaluation of microenvironment • Low predictive negative value
TMB (tumor mutational burden)	Tumors with a higher TMB seem more likely to express neoantigens, inducing a more robust response if treated with CPIs	• Discordant results across different trials • Analysis considered expensive, time-consuming and misleading if performed with an unsuitable NGS panel • No evaluation of microenvironment • Low predictive negative value
Immune cell gene expression profiling	It is considered a comprehensive biomarker that can enable to assess tumor microenvironment and its inflammatory status to distinguish hot tumors from cold ones	• Lack of standardized commercially available gene panel • Expensive • Uncertain negative predictive value of the various gene panel
Granzyme B	It acts as a mediator of target cell apoptosis induced by immune effectors and might be used as a surrogate marker of CD8+ cells activation	• No standardized method of evaluation (levels of soluble marker rather than double staining for CD8+ cells and granzyme B) • Lack of solid data (tried to correlate with response only in a few trials)
DNA damage response (DDR) genes alterations	Association with better response to neoadjuvant chemotherapy and higher TMB and copy number alteration. Plausible a good relation also to CPIs response	• Lack of solid data
Retinoblastoma 1 (RB1) gene alterations	In addition to being fundamental in cell cycle regulation, it has been discovered to be involved in immune function	• Lack of solid data
Epithelial-mesenchymal transition (EMT) markers	In some studies higher EMT-related gene expression was linked to a major benefit from immune checkpoint blockade	• Lack of solid data and contradictory correlations with CPIs response in different studies
TGF-β pathway	It acts as a key factor in cancer development and progression. In some studies high levels of expression were related with resistance to CPIs	• Lack of solid data
Molecular subtyping	Heterogeneous tumors may be grouped by molecular features in several subtypes, different for treatment response and prognosis	• Need for a consensus classification • Need for prospectical trial to validate the retrospective findings about correlation between specific subtypes and different therapy responses

### PD-L1 Expression

Immunohistochemical expression of PD-L1 is the most widely studied biomarker to predict response to immunotherapy in solid tumors. A correlation between higher PD-L1 expression (on tumor cells and/or immune cells infiltrating the tumor) and likelihood of response to CPIs has been seen in particular in melanoma, NSCLC, and renal cell carcinoma (43–45). In several studies concerning bladder cancer, the PD-L1 expression seems to be associated with more advanced pathologic stages at the time of surgical resection and with an increased all-cause mortality, suggesting its potential prognostic role but not supporting its predictive value that still remains uncertain (46–48). In the PURE-01 trial, neoadjuvant pembrolizumab achieved a pCR rate of 42% in all unselected patients and of 54.3% in patients with high PD-L1 combined positive score. By the contrary, the pCR rate was only 13.3% in patients with low PD-L1 expression. Unfortunately, in the ABACUS trial a statistically significant correlation between PD-L1 expression levels and response to neoadjuvant immune CPI was not observed. In the metastatic setting also, the correlation with PD-L1 expression and response to immunotherapy still remains controversial, because several trials observed a response to immune CPIs regardless of PD-L1 expression (14, 49–52). Differences in PD-L1 IHC platforms (different detection antibodies, cell types considered—tumoral and/or immunological cells—and different scores and cut-off definitions for positivity) used in different studies and the dynamic nature of this biomarker over time (different levels expression in different moments of tumor history) and space (mismatch of level expression between primary tumor and metastases) may explain these controversial results, suggesting that PD-L1 expression alone does not seem able to give account of the meaningful of interactions necessary for predicting a T cell response (no evaluation of local effects on the cytokine milieu and immune landscape entirety) (53–55).

### Tumor Mutational Burden

Tumors with a higher TMB seem more likely to express a high number of neoantigens, inducing a more robust response if treated with immune CPIs (56). In the PURE-01 trial, a non-linear association between higher TMB (scores ≥15 mut/Mb) and pCR was found. Likewise, in the IMVigor-210 trial (a phase 2 trial investigating the clinical activity of PD-L1 blockade with atezolizumab in metastatic urothelial carcinoma), the median mutational load was higher in responders to atezolizumab than in non-responders (12.4 mut/Mb vs. 6.4 mut/Mb) (17). Moreover, in the PURE-01 trial, the TMB emerged also as a potential dynamic biomarker of immune CPIs resistance. In fact, comparing pre- and post-therapy TMB levels in patients with pT ≥ 2, a significant decreasing was found (median post-therapy TMB of 5.7 mut/Mb vs. 11.4 mut/Mb in naïve patients), suggesting that pembrolizumab induces selection of less immunogenic neoplastic clones in non-responder patients. Unfortunately, the ABACUS trial did not confirm these findings because no correlation was found between high TMB and increased percentage of response to neoadjuvant atezolizumab. Therefore, the predictive value of TMB still remains controversial, as some immunotherapy responses were noted in patients with lower TMB levels and, in general, even tumors with relatively fewer neo-antigens, such as renal cancer, respond to immunotherapy (54, 57). In addition to these discordant data, the TMB analysis presents other limitations such as the high costs, the time-consuming and misleading if it is performed with an unsuitable Next Generation Sequencing (NGS) panel (53).

### Immune Cell Gene Expression Profiling

The role of the immune microenvironment to predict the response to immunotherapy is becoming clear. In fact, the determination of the immune microenvironment profile discriminates “hot” (potentially responsive to immunotherapy) from “cold” tumors (potentially not responsive to immunotherapy) (58, 59). For this purpose, the evaluations of PD-L1 expression and TMB are not sufficient, while immune cell gene expression profiling is considered a more comprehensive immune biomarker. By using targeted gene expression panels, this integrative analysis may quantify specific RNA expression profiles, enabling to assess the tumor microenvironment and its inflammatory status, by quantifying chemokines, cytokines, cell surface proteins, and molecules involved in T cell signaling and antigen presentation. Together, all these elements may help to identify a “hot” tumor better than PD-L1 expression alone (54, 60). Several gene expression profiling panels to identify a possible predictive role for chemotherapy and immunotherapy response have been investigated in different malignancies, urothelial cancer included (51, 59, 61–63). In the Checkmate 275 trial, pretreatment biopsies of 177 urothelial tumor samples were assessed by using an interferon-gamma 25-gene signature to evaluate a potential relationship between a specific immune microenvironment status and the responsivity to nivolumab. The analysis showed that higher values in the IFN-γ gene signature were correlated with higher percentage of response to nivolumab (33.9% with high IFN-γ signature vs. 16.1% with non-high IFN-γ signature) (51). Similarly, in the ABACUS trial, tGE8, a transcriptional signature of eight genes (IFNG, CXCL9, CD8A, GZMA, GZMB, CXCL10, PRF1, and TBX21), previously described in locally advanced or metastatic urothelial carcinoma, resulted significantly increased in patients responsive to atezolizumab compared to non-responder patients or in patients with disease relapse.

Therefore, gene expression profiling represents a very promising predictive biomarker even if several issues are still open. In fact, the lack of standardized commercially available gene panels as of yet and their uncertain negative predictive value, since some responses were also identified in patients with a non-inflamed cytokine signatures, lead to be cautious (54).

### CD8+ Cells and Granzyme B

In the ABACUS trial, a correlation between the pre-existing T-cell immunity and the response to atezolizumab was observed. In fact, the pCR rate was 40% in tumors characterized by a higher presence of intraepithelial CD8+ cells whereas it was only 20% in tumors without infiltration of CD8+ cells. Moreover, during treatment, an increase of CD8+ cells occurred in responding tumors and not in relapsing ones. The patterns of tumor immune cell infiltration are typically distinguished in: (1) inflamed phenotype, rich in tumor-infiltrating lymphocytes; (2) immune-excluded phenotype, with immune cells around the margin of the tumor but without infiltration inside it; (3) immune-desert phenotype, with absence of relevant numbers of immune cells in tumor tissues (64, 65). However, not all tumors with a consistent immune infiltrate showed a good response to immune CPIs, supporting the idea that the quality of the immune infiltrate, beyond CD8 expression, is relevant in determining outcome.

Granzyme B is a serine protease that acts as a mediator of target cell apoptosis induced by immune effectors such as cytotoxic T lymphocytes. It might be used as a surrogate marker of CD8+ cells activation. In the plasma of patients with NSCLC under treatment with nivolumab, the concentrations of Granzyme B resulted significantly higher in responders rather than non-responder ones, suggesting a stronger activation of the CD8 T cytotoxic immune response (66).

In the ABACUS trial, the tumor samples with an inflamed phenotype were analyzed for dual CD8+ cells and Granzyme B staining, founding that responding tumors expressed more cells dually stained compared to relapsing ones (87 vs. 30% of dual expression). Moreover, after neoadjuvant atezolizumab, a significant increase in intraepithelial CD8 expression (78% increase in median values) and in Granzyme B was observed. Although a similar analysis has been only tested so far in this study, it might be considered and tested in further trials to confirm the predictive role of Granzyme B levels regarding the response to immunotherapy.

### DNA Damage Response (DDR) Genes Alterations

Mutations in this family of genes are known to be associated with tumor development, progression, treatment response, and outcome in urothelial cancer (67). In bladder cancer, tumors carrying DDR genes alterations achieved a higher ORR to cisplatinum-based neoadjuvant chemotherapy (68). The association of DDR genes alterations with a higher mutational burden and increased gene copy number alterations justifies a better responsivity to immune CPIs, as observed in several solid tumors (69–71). Unfortunately, the data supporting the predictive role of DDR gene mutations about response to immunotherapy in bladder carcinoma are still insufficient. Moreover, in the ABACUS trial, alteration status in DDR-related genes, observed at baseline and stratified by outcome, did not show a significant correlation with response to atezolizumab.

### Retinoblastoma (RB1) Gene Alterations

The *RB1* gene is a fundamental cell cycle regulator, whose inactivation is notoriously associated with cancer development (72). Emerging data also suggests its involvement in immune function, with the observation, in RB1-loss models, of a reduced expression of many factors involved in immune response (such as surface receptors, complement, lymphocyte factors, and cytokines) (73). Moreover, phosphorylated RB1 seems to interact with nuclear factor kB (NF-kB) inducing a downregulation of NF-kB transcriptional targets, including PD-L1. All these elements suggest a potential role of RB1 in immunotherapy response and in predicting it. In NSCLC patients, RB1 alterations have been correlated with the lack of response to second line nivolumab or first line pembrolizumab (74). Similarly, in non-MIBC, the RB1 under-expression has been related to non-response to immunotherapy with intravesical bacille Calmette-Guerin (BCG) and with tumor recurrence (75–77). However, in the PURE-01 trial, the apparently strong association between DDR and/or RB1 genes alterations and response to immunotherapy (increasing of pCR rate up to 60% in patients with tumors carrying these alterations compared to 39.5% observed in unselected patients) resulted weakened when adjusted for TMB. Therefore, further data are needed to explore the role of *RB1* gene alterations.

### TGF-β Pathway

TGF-β is a pleiotropic cytokine, which acts as a key factor in cancer development and progression, by promoting immunosuppression, angiogenesis, epithelial mesenchymal transition (EMT), and metastases development (78, 79). By analyzing molecular features of responder patients compared to non-responder ones in the population enrolled in the IMvigor210 trial, Mariathasan and collegues found that TGF-β gene expression was significantly associated with the resistance to immunotherapy and with a shorter overall survival, especially in the presence of the “excluded tumor-immune phenotype” (80). Further studies are needed to confirm these data.

### Molecular Subtyping

The molecular subtyping might be an effective tool to personalize immunotherapy use. Since the 1990s, cytogenetic and karyotype studies have started suggesting the importance of biomolecular features in this type of neoplasm corroborating that bladder carcinoma is a heterogeneous disease. (81). Advances in genomic sequencing have enabled to perform increasingly sophisticated genomic analyses on large cohorts of bladder cancer samples, with the aim of classifying such a molecularly heterogeneous cancer into specific genomic subtypes with similar biomolecular features, prognosis and response to treatment, especially in a view of personalized medicine. In 2014, The Cancer Genome Atlas (TCGA) research group provided a first comprehensive molecular characterization of 131 high-grade MIBC, studied by multiple platforms, in order to analyze whole genome and exome sequencing, copy number variations and expression of mRNA, protein, miRNA and long non-coding RNA (82). On the basis of these analysis and especially of mRNA expression, four clusters were initially identified, but, in a subsequent analysis of 412 MIBC, published in 2017, the taxonomy of MIBC was expanded, identifying five molecular subtypes: (1) luminal-papillary (35%), enriched in mutation or amplification of FGFR3 or FGFR3 gene fusions with TACC3; (2) luminal-infiltrated (19%), with overexpression of extracellular matrix and smooth muscle gene and of immune markers, such as PD-L1 and CTLA-4 and with high level of immune infiltrates; (3) luminal (6%), with high expression of uroplakin genes, KRT20 and SNX31; (4) basal-squamous (35%), with high expression of basal marker genes (such as KRT5, KRT6A, and CD44), squamous differentiation markers, immune expression genes (such as CXCL11 and L1CAM) and TP53 mutations; (5) neuronal (5%), with high expression of neural differentiation and development genes (such as SOX2, MSI1, and GNG4) and high level of alterations in genes of the p53/cell-cycle pathway (83). This analysis reinforces the idea that different molecular subtypes could benefit from different clinical options. The luminal-papillary subtype, usually characterized by papillary histology and low CIS scores, was seen having a low risk for progression but also a low likelihood of response to cisplatin-based NAC. Therefore, an anti-FGFR3 target therapy could be more useful than the standard chemotherapy. The neuronal subtype, presenting molecular features similar to lung squamous cell carcinoma, could achieve more benefit from a chemotherapy with platin-etoposide than standard chemotherapy with cisplatin and gemcitabine. In the same way, luminal-infiltrated subtype, that appears to be resistant to cisplatin-based chemotherapy, has been reported to respond to immune CPIs in patients with metastatic or unresectable bladder cancer. Instead, basal-squamous subtype seems to benefit both from cisplatin-based NAC and from immune CPIs (83). Song et al. (84) identified four distinct molecular subtypes of BC (displaying discriminative biological and clinical features, even considering all pathological subtypes) using a total of 1,934 samples from seven different cohorts of patients carrying not only MIBC but also NMIBC, including a subgroup containing progressive NMIBC and MIBC with poor prognosis that would benefit from ICIs therapy. This specific subgroup presented distinct features of high mutation load, inhibited TGFβ signaling, and activated cell cycle. In particular, they identified that patients with this specific BC subtype were significantly responsive to an anti-PD-L1 agent in the IMvigor210 cohort. In general, in addition, other molecular classifications have been formulated in the last 10 years. Although there is a significant overlapping with each other in the obtained results, a consensus about a specific classification and its validation in prospective trials are needed (85).

In conclusion, the recent published data on the activity of immune CPIs have changed the contemporary treatment landscape for patients with metastatic urothelial carcinoma. Important benefits from immunotherapy are awaited also in perioperative setting and the few available data have showed promising results (PURE-01 and ABACUS trials). The confirmation of the efficacy of the perioperative immunotherapy by the ongoing trials, the added advantage of its better toxicity profile compared to cisplatin-based chemotherapy, and the potential availability of biomarker-driven approaches might change in the near future the clinical practice and expand the sample of treatable patients without compromising their quality of life.

## Author Contributions

FDA, FB, NC, and PZ: study conception, design and drafting of manuscript, and analysis and interpretation of data. FDA, FB, and NC: acquisition of data. FDA, FB, NC, MP, FDV, AS, and PZ: critical revision. FDA, FB, NC, MP, FDV, AS, and PZ: final approval. All authors contributed to the article and approved the submitted version.

## Conflict of Interest

The authors declare that the research was conducted in the absence of any commercial or financial relationships that could be construed as a potential conflict of interest.
